# Optimization of the Cetyltrimethylammonium bromide (CTAB) DNA extraction protocol using forest elephant dung samples

**DOI:** 10.1016/j.mex.2022.101867

**Published:** 2022-09-19

**Authors:** Jean-Louis Kouakou, Sery Gonedelé-Bi, Jean-Baptiste Assamoi, Simon-Pierre Assanvo N'Guetta

**Affiliations:** a Laboratoire de Biotechnologie, Agriculture et Valorisation des Ressources Biologiques, Université Félix Houphouët-Boigny d'Abidjan-Cocody, Côte d'Ivoire; 22, BP 582 Abidjan, Côte d'Ivoire; b Centre Suisse de Recherches Scientifiques en Côte d'Ivoire; 01 BP 1303 Abidjan 01, Côte d'Ivoire

**Keywords:** Non-invasive sample, PCR success, DNA quality and quantity, Improved CTAB method, Fecal sample

## Abstract

Among non-invasive biological samples, feces offer an important source of DNA and can easily be collected. However, working with fecal DNA from highly vegetarians species such as elephant is more challenging because plant secondary compounds have an inhibitory effect on PCR reactions.

Working with forest elephant dung samples, we tested and adapted a protocol of DNA extraction developed on plants based on the Cetyltrimethylammonium bromide (CTAB) protocol.

The protocol is relatively simple and yields a high DNA concentration. It is five-time less expensive compared to the methods of Benbouza et al. The extracted DNA is of good quality and easily amplified by PCR. The high-amplification percentage of mitochondrial genes in fecal DNA and subsequent sequencing of PCR products indicate that the proposed optimized method is reliable for molecular analysis of forest elephant dung samples.

• Our optimized CTAB protocol has been adjusted by the addition of Sodium Dodecyl Sulfate (SDS) and proteinase K during the lysis phase. The combined effect of these reagents was capable of lysing cell walls and removing proteins efficiently.
• Moreover, the prolonged time of incubation (overnight incubation at room temperature followed by 3 hours of incubation in a water bath) enhanced the increase of DNA yield but make the optimized protocol more time-consuming.

## Graphical Abstract

**Figure:**
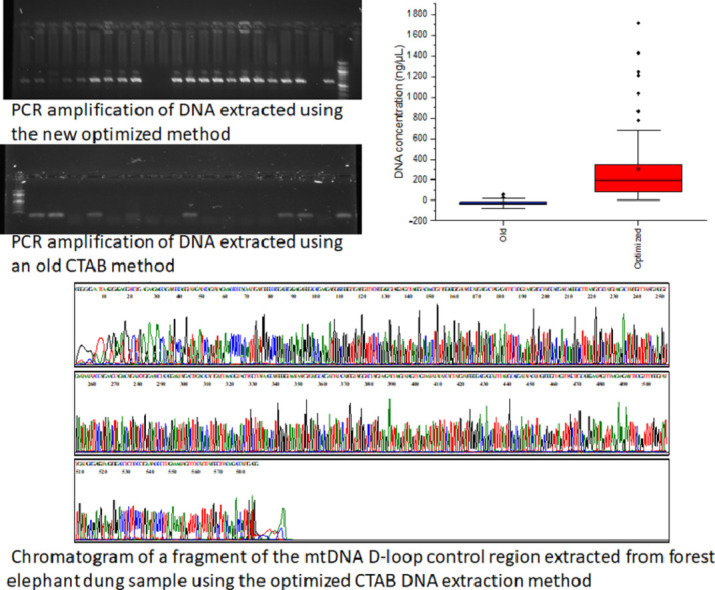

## Specifications Table

**Table:** 

Subject Area:	Biochemistry, Genetics and Molecular Biology
More specific subject area:	DNA extraction
Method name:	Cetyl Trimethyl Ammonium Bromide (CTAB) DNA extraction protocol
Name and reference of original method:	H. Benbouza, B. Jean-Pierre, G. Mergeai, Amélioration de la méthode d'extraction d'ADN au CTAB appliquée aux feuilles de cotonnier. Biotechnologie, agronomie, société et environnement. 10 (2006) 73‑76.
	J.J. Doyle, J.L. Doyle, Isolation of plant DNA from fresh tissue. Focus. 12 (1990) 39‑40.
	M.G. Murray, W.F. Thompson, Rapid isolation of high molecular weight plant DNA. Nucleic acids research. 8 (1980) 4321‑26.
Resource availability:	n/a

## Method details

### Background

Noninvasive techniques have been largely used in ecology for molecular studies because they present the advantage to not disturb or arm the studied animals [1]. Among non-invasive biological samples, feces offer an important source of DNA and can easily be collected.

Several methods have been proposed to extract DNA from feces, such as the guanidinium thiocyanate silica method [2], [3], [4], the aqueous two-phase system method [5], the phenol-chloroform method [6], the Chelex 100 method [7,8], and the washing technique [9]. The application of these methods is however limited, especially for herbivorous species. Several limitations are presented by different methods actually used for DNA isolation. These include the presence of inhibitors, the quality of feces, the amount of feces used, and the preservative fluids [9]. Commercial kits offer efficient and convenient alternatives but are comparatively expensive, and sometimes fail in samples from herbivorous animals. Because, any single DNA extraction technique is efficient for all species, various techniques targeting different species have been proposed [10].

Working with fecal DNA from highly vegetarians species such as elephant is more challenging. Indeed, plant secondary compounds have an inhibitory effect on PCR reactions. PCR success then depends on the maximization of DNA concentration, while reducing secondary compounds derived from plants in the diet [11,12].

Such a low amplification success reduces considerably the suitability of fecal samples for genetic studies in herbivores. Therefore, it is important to optimize the genetic techniques to obtain an adequate amplification success rate.

CTAB method has been widely applied in molecular genetics of plants [13]. That detergent-based method has already been tested in some studies based on non-invasive samples [14,15]. Several other variations have been developed with the aim of adapting the method to a large number of organisms [16,17].

An alternative combining at the same time a low cost, a saving of time, a guarantee of good quality DNA extraction, and the use of non-harmful reagents not requiring special handling devices must therefore be sought.

Working on forest elephant, we tested and adapted a protocol developed by Vroh Bi et al. [18] on plants based on the CTAB (cetyltrimethylammonium bromide) protocol of Murray & Thompson [19] described by Chandellier [20]. CTAB is a frequently used surfactant in DNA extraction and several modifications of that protocol originally published by Doyle and Doyle [21] have been used.

To optimize the quantity and quality of DNA extracted from forest elephant dung samples, known for its high essential oil and polyphenolic content [22], [23], [24], different parameters including optimizing tissue lysis phase, DNA precipitation, duration of incubation time, duration of DNA drying and temperature of DNA storage have been tested to maximize the amount and quality of DNA retrieved.

The standard extraction protocol is that of Doyle et al. [21] based on the cationic detergent Cetyltrimethylammonium bromide (CTAB) previously used by Murray and Thompson [16] and Benbouza et al. [25]. These authors used that protocol to respectively extract DNA from leaves of various species (wheat, peas, oats, carrots, mung beans, tobacco, Mimulus sp., Atriplex sp., and cotton). Adjustments were made based on the proposed method of Benbouza et al. [25] (Table 1).

**Table 1:** Comparison of the main steps of the DNA extraction methods using the CTAB method proposed by Benbouza et al. [25] and our optimized protocol.

Benbouza et al. [25]	New optimized method
**Buffers**	
2 % hexadecyl trimethyl ammonium bromide (CTAB), 2 % polyvinylpyrrolidone, 2.0 M NaCl, 20 mM EDTA, 100 mM Tris-HCL (pH 8.0) and 5% ß-mercaptoethanol	2 % CTAB, 1.4 M NaCL, 10 mM 0.5 M EDTA and 100 mM 1 M Tris HCL (pH 8.0)
**Biological material**	
Cotton leaves	Forest elephant dung
**Sample lysis**	
Addition of lysis buffer to CTAB and mixed well by vortexing.	Addition of lysis buffers to CTAB, Sodium Dodecyl Sulfate (SDS) (20%) and proteinase K (20 mg/mL) well mixed by vortexing.
	Incubate for 24 h at room temperature.
Incubate for 1 hour at 65°C.	Incubate in a water bath for 3 h at 65°C.
**DNA precipitation**	
Centrifuge for 10 min at 16,300 rotations per minute (rpm) at 4°C.	Centrifuge for 1 min at 13,000 rpm at 26°C.
	Transfer of the supernatant and addition of 400 μL of chloroform.
	Centrifuge for 5 min at 13,000 rpm at 4°C.
**DNA washing**	
Transfer of the supernatant and addition of 2/3 of the withdrawn volume of isopropanol stored at 4°C.	Transfer of the supernatant and addition of 500 µL of isopropanol.
	Incubate the solution at 4°C for 15 min.
Centrifuge for 5 min at 13,000 rpm at 4°C.	Centrifuge for 15 min at 13,000 rpm at 4°C.
Transfer the supernatant and add 10 ml of wash buffer per g of crushed leaves.	Add 500 µL of 70°C ethanol to the pellet, mix by inversion and incubate for 5 min at room temperature.
Centrifuge for 5 min at 13,000 rpm at 4°C.	Centrifuge for 5 min at 13,000 rpm at 4°C.
**Drying the DNA pellet**	
Air dry the DNA for 20 min.	Dry the pellet for 30-60 min at room temperature.
**Solubilization**	
Suspend the DNA pellet in 100 to 300 μl of TE 1x and add 8 μl of RNase per 100 μl of DNA.	Add 50-100 µl of ultra-pure water to each pellet.
Store DNA extracts at 4°C.	Store DNA extracts at -20°C.

### Biological sample: elephant dung samples

From 07 to 30 October 2019, dung samples of forest elephant were collected at stage 1 of decomposition (dung pile intact, very fresh, and moist, with odour) in the Bossématié, Dassioko and Port-Gauthier Forest Reserves respectively located in the rainforest area in Côte d'Ivoire. Each fecal sample was preserved in 50 ml cryotube containing 70 % ethanol. All samples were transported to the laboratory within 15–20 days and stored at -80°C till extraction.

### Reagents and solutions

The reagents and chemicals used are extraction buffer (CTAB, NaCl, 1M Tris-HCl, EDTA.Na), chloroform: isoamylalcohal (24:1), absolute ethanol, 70% ethanol, TE (1M Tris-HCl, 0.5M EDTA.Na) buffer pH 8.0 and TBE (Tris, Boric acid, EDTA.Na).

*Procedure for extracting DNA from dung samples*

A. Preparation of 2 % CTAB lysis buffer and solutions

**Table:** 

Reagents	Volume	Final concentration
1 M Tris HCL pH 8.0	100 ML	100 mM
0.5 M EDTA	20 ML	10 mM
NaCL	81.8 g	1.4 M
CTAB	20 g	2 %
Distilled water	QSP 1L	

Mix under a magnetic stirrer.

Store the buffer at room temperature.

Clean the work area with bleach or alcohol and change gloves between samples.

B. Lysis of fecal sample

1. Add 150 mg of dried fecal sample to 1.5 ml Eppendorf tubes or if the sample is liquid, shake the tube containing the sample vigorously, remove 300 µL of the fecal suspensions, and pour it into 1.5 mL Eppendorf tubes.
Note: if the sample is liquid, cut the tip of the pipette cone to facilitate pipetting.
2. Add to each sample successively, 1000 µL of lysis buffer, 50 µL of Sodium Dodecyl Sulfate (SDS) (20 %), and 20 µL of proteinase K (10 mg/mL).

Optional: add 4 µL of RNase A to each sample.

3. Mix the solution by vortexing for 1 min.
4. Incubate all samples at room temperature for approximately 24 h or overnight.
5. The next day, mix the solution by inversion for 3 min.
6. Incubate all samples in a water bath for 3 h at 65°C.
7. Centrifuge all samples for 1 min at 13,000 rotations per minute (rpm) at 26°C.

C. DNA precipitation

8. Take 1000 µL of the supernatant from the solutions of each sample (the upper phase) and place it in another 1.5 mL Eppendorf tube.
9. Add to each sample 400 µL chloroform.
10. Mix solutions gently by inversion for 3 min.
11. Incubate all samples for 15 min at 4°C.
12. Centrifuge all samples for 5 min at 13,000 rpm at 4°C.

13. Take 900 µL of the supernatant from each sample (the upper phase) (avoid contact with the white layer) and place it in another 1.5 mL Eppendorf tube.

D. DNA purification

14. Add 500 µL of isopropanol to all samples.
15. Mix gently by inversion for 3 min.
16. Incubate the solution mixture in the refrigerator at 4°C for 15 min.
17. Centrifuge for 15 min at 13,000 rpm at 4°C.
18. Remove the isopropanol, drain well.
19. Leave to dry at room temperature for 10 min.
20. Add 500 µL of 70°C ethanol.
21. Mix well by inversion for 3 min.
22. Incubate all samples for 5 min at room temperature.
23. Centrifuge for 5 min at 13,000 rpm at 4°C.
24. Drain well the 70°C ethanol.
25. Dry the pellet for 30-60 min at room temperature.

E. Solubilization

26. Add 50 to 100 µl of high purity water to each pellet.
27. Incubate the DNA extract obtained for 5 min in a water bath at 65°C.
28. Peel off the caps by vortexing and
29. Store DNA extracts at -20°C.

### Determination of the quantity and purity of the extracted DNA

A volume of 1 μl of each DNA extract was used for DNA quantification using a Thermo Scientific Nanodrop spectrophotometer (*NanoDrop 2000 C, USA*) by calculating the 260/280 and 260/230 absorbance ratio of each sample [26]. The integrity of the DNA was checked by 1 % agarose gel electrophoresis.

### Validation of DNA quality and integrity using PCR and sequencing

Primer pairs LafCR1 F: 5′-GTATAAGACATTACAATGGTC-3′(10μM) and LafCR2 R: 5′-AGATGTCTTATTTAAGAGGA-3′ [18] were used to amplify approximately 600 base pairs (bp) of the mtDNA segment located in the hypervariable I of the control region. Polymerase chain reaction (PCR) was performed in a total volume of 50 mL reactions containing 1 μl of genomic DNA (317.027 ± 338.982 ng/µL), 25 μl Quick-Load one Taq 2X Master Mix with Standard Buffer (*New ENGLAND Biolabs, USA*), 1 μl of Dimethyl sulfoxide (10 %) (*Eurolabs, France*), 1 μl of magnesium chloride (MgCl2) (*New ENGLAND Biolabs, USA*) (25 mM), 4 μl of Bovin serum albumin (BSA) (SIGMA-ALDRICH, Germany) (10%), 1 μl of each primer (10 μM) and 15 μl volume Ultrapure water (H_2_O). This mixture was processed in the thermal cycler Techne TC-512 for PCR reactions at different successive temperature cycles. A total of 40 cycles were performed. Each cycle consists respectively of an initial denaturation at 95°C for 5 min, followed by denaturation at 95°C for 30 s, a hybridization phase at 38.4°C for 45 s, and an elongation at 72°C for 30 s, then a final termination cycle at 72°C for 5 min and finally a cooling phase at 4°C.

DNA sequencing was performed by BGI BIO-SOLUTIONS HONGKONG CO., LIMITED company following the techniques of Sanger et al. [27].

### Validation of the optimized CTAB method

**Fig. 1:**
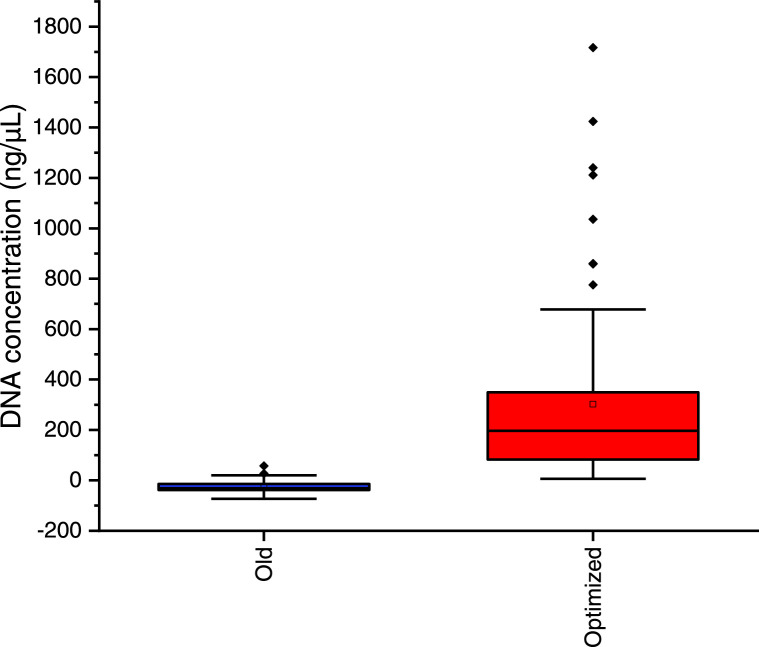
Boxplot of the quantity () of the DNA extract obtained based on the method of Benbouza et al. [25] and the optimized method using forest elephant dung samples stored in 70 % ethanol.

Of the 97 dung samples preserved with 70 % ethanol, 6.2 % (6/97) yield DNA when using the extraction method of Benbouza et al. [25], whereas DNA was successfully obtained from all 97 fecal samples (100 %) using the proposed optimized method. That later yields a mean DNA concentration (317.027 ± 338.982 ng/µL) higher than that obtained with the method of Benbouza et al. [25] (-24.278 ± 19.938 ng/µL) (*U*-test of Mann-Whitney, *p*-value = 0.0001 <0.05) (Fig. 1).

**Fig. 2:**
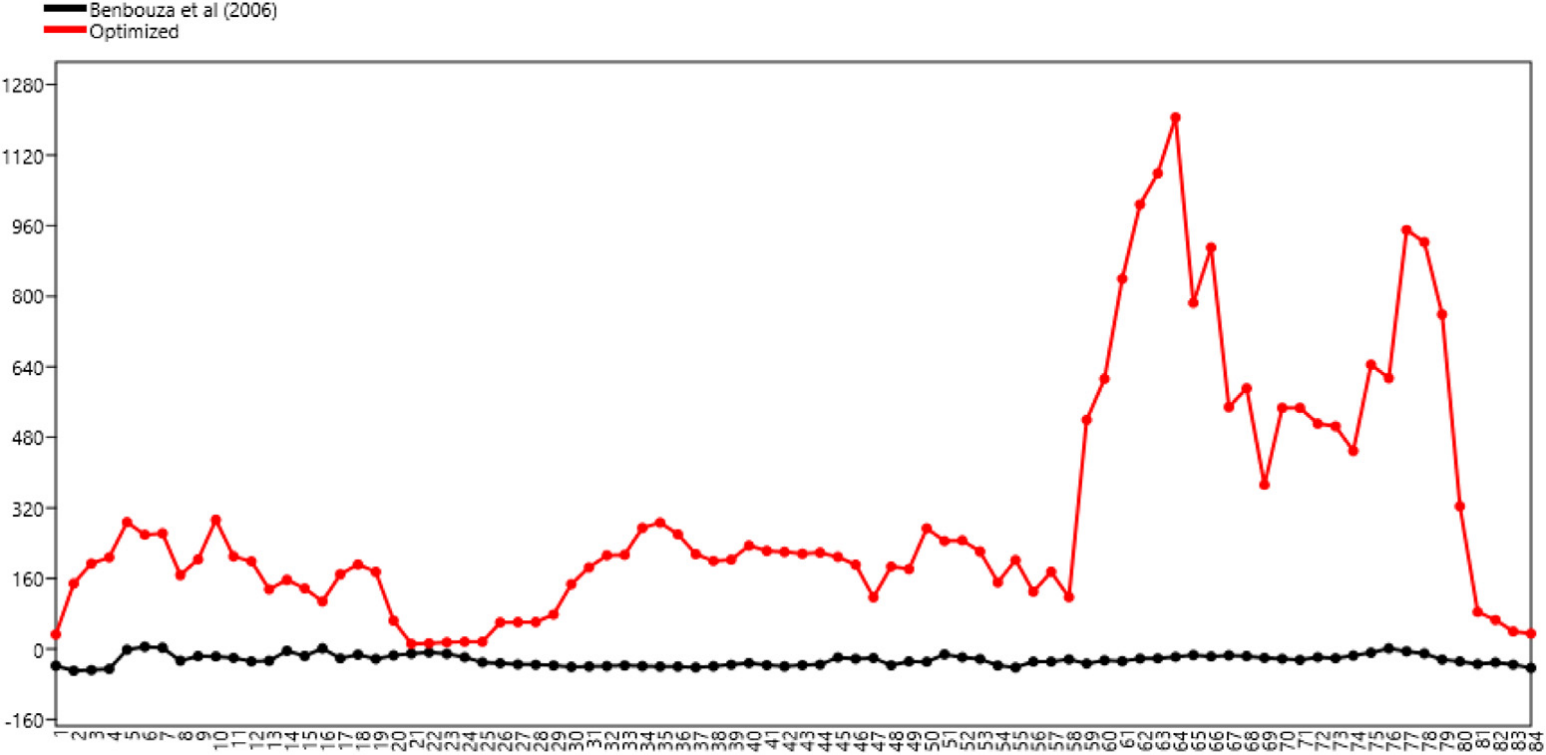
Percentile curve of DNA concentration obtained from samples of forest elephant dung stored in 70 % ethanol and extracted with the old method of Benbouza et al. [25] and the new optimized method.

The delimitation of the two methods is also presented by the percentile profile (Fig. 2). The all total DNA samples extracted using the optimized method yields over 5 ng/µL whereas the majority of the samples extracted using the method of Benbouza et al. [25] yields less than 0 ng/µL, exception for six samples yielding from 0.3 to 26.9 ng/µL.

**Fig. 3:**
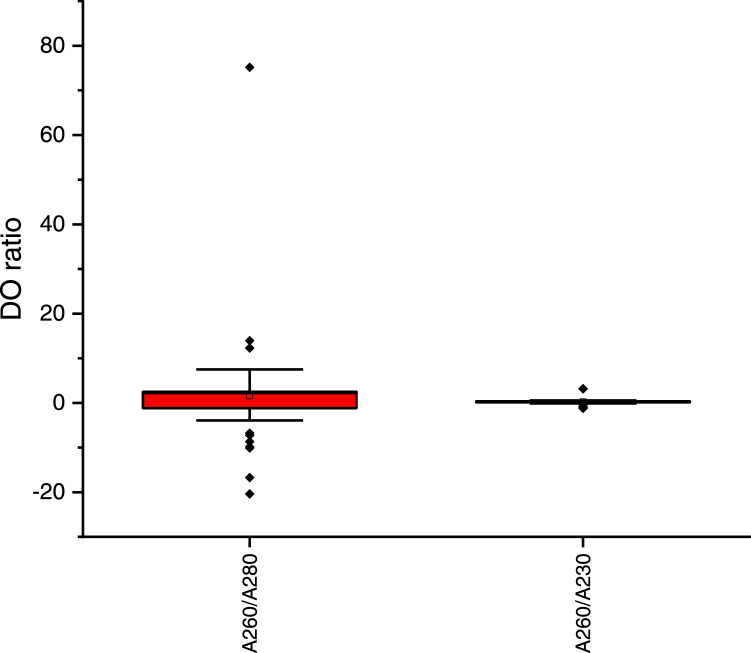
DO ratio of DNA samples extraction using the method of Benbouza et al. [25].

**Fig. 4:**
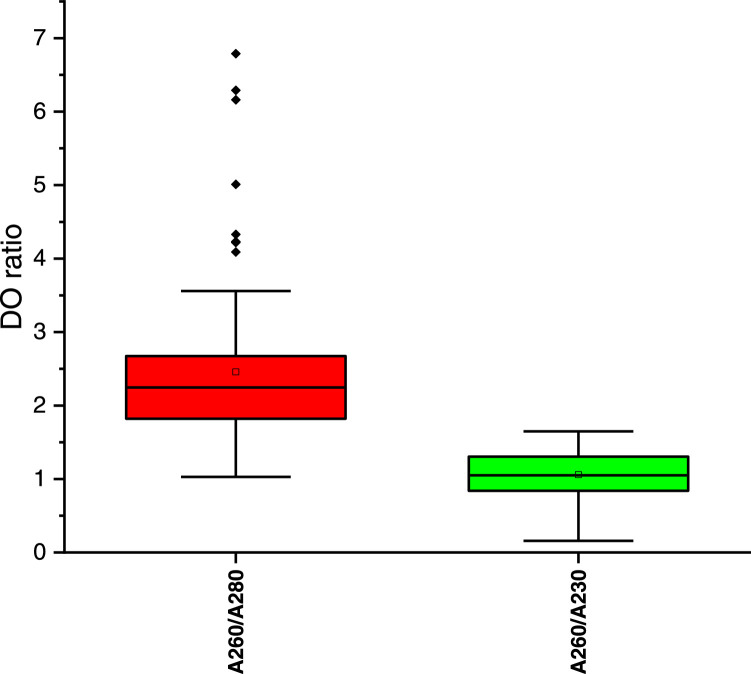
DO ratio of DNA samples extraction using the optimized methods.

The 260/280 and 260/230 ratios of the method of Benbouza et al. [25] were not significantly different (*U*-test of Mann-Whitney, *p* = 0.0001 < 0.05), indicating low yield and the presence of high levels of contaminants (Fig. 3). On the other side, the 260/280 and 260/230 ratios of the optimized method were close to 2.25 and 1.0, respectively (Fig. 4). The 260/280 ratio of the optimized method showed a larger variance.

**Fig. 5:**
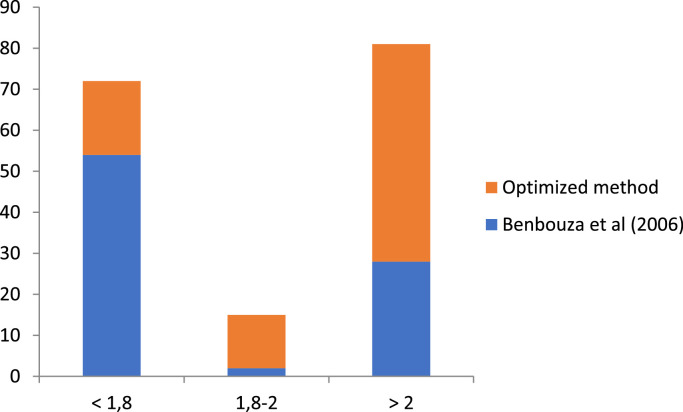
Graph of 260/280 absorbance ratios (DNA quality parameters) for the optimized methods of DNA extraction.

The large majority (65%) of the sample extracted using the method of Benbouza et al. [25] were out of the range of good quality DNA (1.8-2.0), indicating a poor quality DNA (Fig. 5). The optimized method gave a relatively higher (14) number of sample in the range of good quality DNA or greater than 2 (Fig. 5).

Of the 97 dung samples extracted with the new optimized method, the amplification success rate was 82.50 % (80 out of 97 samples).

**Fig. 6:**
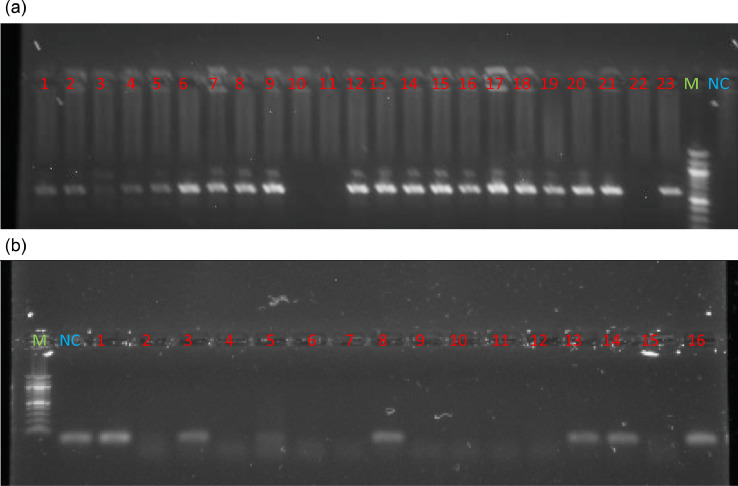
a. PCR amplification of the mitochondrial DNA D-loop region using total DNA extracted from forest elephant dung sample using the new optimized method. Lane 1: molecular weight marker; Lane NC: negative control; Lane 1,2,4-9,12-21 and 23: positive PCR using primer pair LafCR1_F/LafCR2_R b. PCR amplification of the mitochondrial DNA D-loop region using total DNA extracted from forest elephant dung sample using an old CTAB DNA extraction method. Lane 1: molecular weight marker; Lane NC: negative control; Lane 1 to 16: negative PCR showing LafCR1_F/LafCR2_R primer dimer

The results showed that DNA samples extracted by the novel improved methods presented good amplification as proved by clear and thick bands of expected amplicons in the gel, indicating that the improved method resulted in good quality DNA of sufficient quantity (Fig. 6a). The PCRs with DNA extracted using the method of Benbouza et al. [25], fail to amplify, indicating poor quality DNA. The absence of amplicons with the negative control indicates that there was no cross contamination (Fig. 6b).

**Fig. 7:**
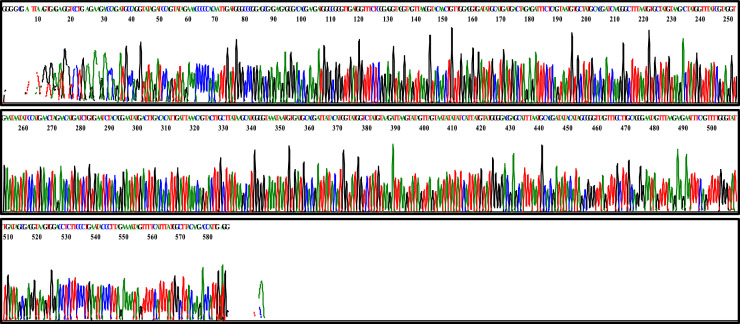
Chromatogram of a fragment of the mtDNA D-loop control region extracted from forest elephant dung sample using the optimized CTAB DNA extraction method.

Of the 80 samples that had a positive PCR, 67.5% (54 out of 80) were successfully sequenced. Approximately 600 base pairs (bp) located in the control region of mtDNA were amplified with primer pairs LafCR1_F and LafCR2_R. The resulting chromatogram depicted evenly-spaced peaks, each with only one color (Fig. 7).

The resulting fragments sequenced were blasted against the NCBI database and the sequences had a 99 % similarity with previously reported sequences of *Loxodonta cyclotis*.

### Estimation of the cost and time of DNA extraction

The time and the cost required to extract DNA from a sample using each of the two methods was determined by summing the times consumed by all the steps of each extraction protocol (Table 2). The New optimized method is approximately eightfold more time-consuming (29 h 45 min) compare to the methods of Benbouza et al. [25]. The average cost of DNA extraction from a sample based on the optimized methods (USD 0.483) is fivefold less expensive compared to the methods of Benbouza et al. [25] (USD 2.697).

**Table 2:** 

Reagent/Plastics	Benbouza et al. [25]	New optimized method	Bulk cost ($ US)	Cost Per Sample ($ US) Benbouza et al. [25]	Cost Per Sample ($ US) optimized method
2% CTAB			$170/1 kg	0.00264	0.00264
NaCl			$43.6/5 kg	0.0021	0.0021
EDTA			$261/1 kg	0.014	0.014
Tris-HCL			$85/1 kg	0.0245	0.0245
5% ß-mercaptoethanol			$19.8/100 mL	0.0099	-
2% Polyvinylpyrrolidone			$55.80/100 g	0.011	-
20% SDS			$58.35/500g	-	0.001167
Ethanol			$12.9/500 mL		0.0000258
proteinase K			$153/10ml	-	0.306
Chloroform			$18/2.5 L	-	0.00288
Isopropanol			$11/2.5 L	0.0022	0.0022
RNase			$103 / 1000U	0.103	-
Wash buffer			$30 / 25 mL	2.4	-
Ultra pure water			$74.05/ 10L	-	0.0074
TE			$26.90 / L	0.00807	-
Plastics				0.12	0.12
TOTAL ($US)				2.69741	0.4829128

## Conclusions

Molecular genetic analyzes in elephant populations are sometimes limited by the availability of DNA extraction protocol, fresh animal material, and the time required for extraction as well as by the quality of DNA extracted from dung samples.

Here we proposed an optimized CTAB DNA extraction protocol using forest elephant dung samples. The protocol is relatively simple and it yields a high DNA concentration. The extracted DNA is of good quality and easily amplified by PCR. The high-amplification percentage of mitochondrial genes in fecal DNA and subsequent sequencing analysis for PCR products indicate that the proposed optimized method is reliable for molecular analysis of forest elephant dung samples. It also has the advantage to be cost-effective and is five times less expensive compared to the method of Benbouza et al. [25]. It also offers significantly easier to prepare and transport samples, and thus is suitable for studies in countries where elephants occur. However, it has the disadvantage of being time-consuming. The results of the present fecal DNA studies could help to assess and evaluate conservation or management policies for forest elephant, a critically endangered species.

## Sources of funding

This research did not receive any specific grant from funding agencies in the public, commercial, or not-for-profit sectors.

## Additional information

The optimized CTAB DNA extraction method using forest elephant dung samples yields DNA concentrations ranging from 6.2 to 1716.9 ng/μL, far greater than the one derived from the method of Benbouza et al. [25] that ranged from -49.4 to 16.3 ng/μL. In addition to yield a high DNA concentration, our optimized method presents a relatively good DNA quality and is more appropriate. Our method amplified the mtDNA of 80 % of elephant fresh dung samples (n = 94). Compare to the study of Renan et al. [28] who were able to amplify mtDNA from 61 % of swab samples, our optimized DNA extraction method is more efficient. Renan et al. [28] had 100 % amplification success for mtDNA using the frozen swab combined with a QIAamp DNA. Using the CTAB extraction method, the mtDNA amplification rate obtained by these authors dropped to 50 % when extracting DNA from swabs taken from fresh samples frozen in the field (n = 8). Hence, when comparing our optimized CTAB method to the CTAB procedure used by these authors, our method has a greater amplification success.

The high-amplification and sequencing percentage of mitochondrial genes in elephant dung sample confirmed that the new optimized method is an efficient and reliable protocol for fecal DNA isolation.

Hence, our method is a good option to conduct molecular research on forest elephant dung samples.

The difference in performance observed between the proposed optimized methods and the one proposed by Benbouza et al. [25] could be explained by the efficiency of the optimized CTAB/SDS/Proteinase K procedure to lyse cell walls more easily [29], [30], [31], [32] than the methods of Benbouza et al. [25].

SDS is a key component very well for cell lysis [33,34], and is the basis of a very well–established method for protein purification and molecular weight estimation [35,36].

The prolonged incubation time (overnight incubation at room temperature followed by 3 h of incubation in a water bath) has certainly increased the DNA yield but prolonged eightfold DNA extraction time. The effect of incubation time and other parameters such as the temperature needs further evaluation since several studies [37], [38], [39], [40] indicated that the incubation time differentially impacts on DNA yield depending on the type of sample or the preservation condition used.

## Declaration of Competing Interest

The authors declare that they have no known competing financial interests or personal relationships that could have appeared to influence the work reported in this paper.
